# Supplementary material for: Rewiring mitochondrial phosphatidylethanolamine metabolism identifies new and unaccounted trafficking steps
Source: J Lipid Res. 2026 Jun 19;67(8):101083. doi: 10.1016/j.jlr.2026.101083 (PMC13425903; doi:10.1016/j.jlr.2026.101083)
Supplement: Supplemental Figure S1 [file mmc1.docx]

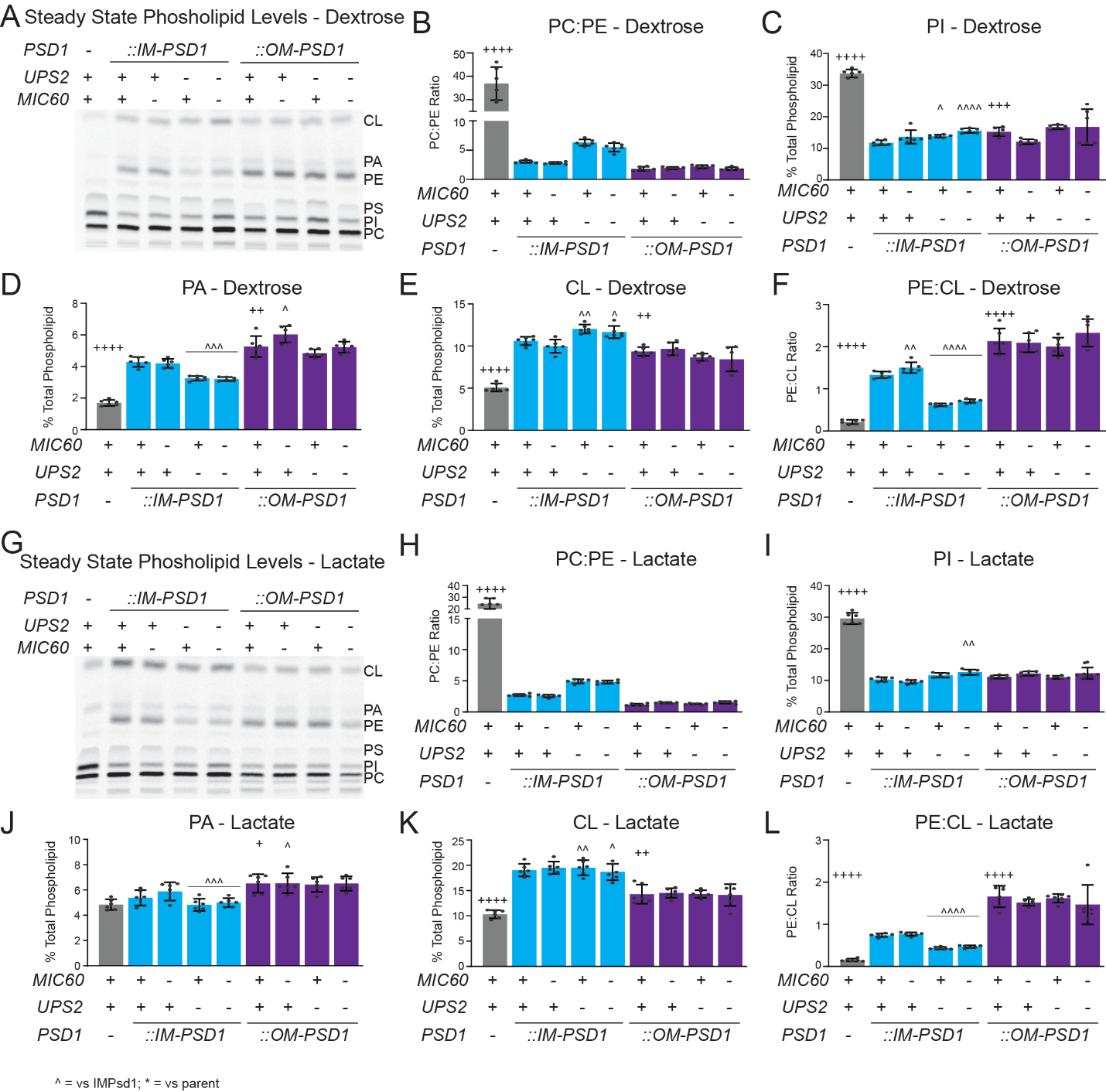


Figure S1. OM-Psd1 short-circuits the need for Ups2 or Mic60. Mitochondrial phospholipids were labeled overnight with ^14^C-acetate in indicated yeast grown in rich (A) dextrose or (G) lactate medium and resolved by TLC (different representative TLC images versus those shown in Fig. 2, E and F, are provided for each). Quantitation of mitochondrial PC:PE ratio (B, H), PI (C, I), PA (D, J), CL (E, K) amounts and PE:CL ratio (F, L) (mean ± SD for *n* = 6 biological replicates from 2 clones/genotype). Significant differences compared to the respective Ups2- and Mic60-proficient parent (^) or IM-Psd1 (+) were determined by one-way ANOVA with Tukey’s multiple comparisons.
